# Illuminating the Transcriptome through the Genome

**DOI:** 10.3390/genes5010235

**Published:** 2014-03-14

**Authors:** David J. Elliott

**Affiliations:** Institute of Genetic Medicine, Newcastle University, Newcastle, NE1 3BZ, UK; E-Mail: David.Elliott@ncl.ac.uk; Tel.: +44-0191-241-8694; Fax: +44-0191-241-8666

**Keywords:** transcriptome, RNA splicing, post-genome

## Abstract

Sequencing the human genome was a huge milestone in genetic research that revealed almost the total DNA sequence required to create a human being. However, in order to function, the DNA genome needs to be expressed as an RNA transcriptome. This article reviews how knowledge of genome sequence information has led to fundamental discoveries in how the transcriptome is processed, with a focus on new system-wide insights into how pre-mRNAs that are encoded by split genes in the genome are rearranged by splicing into functional mRNAs. These advances have been made possible by the development of new post-genome technologies to probe splicing patterns. Transcriptome-wide approaches have characterised a “splicing code” that is embedded within and has a significant role in deciphering the genome, and is deciphered by RNA binding proteins. These analyses have also found that most human genes encode multiple mRNA isoforms, and in some cases proteins, leading in turn to a re-assessment of what exactly a gene is. Analysis of the transcriptome has given insights into how the genome is packaged and transcribed, and is helping to explain important aspects of genome evolution.

## 1. Introduction

**Figure 1:**
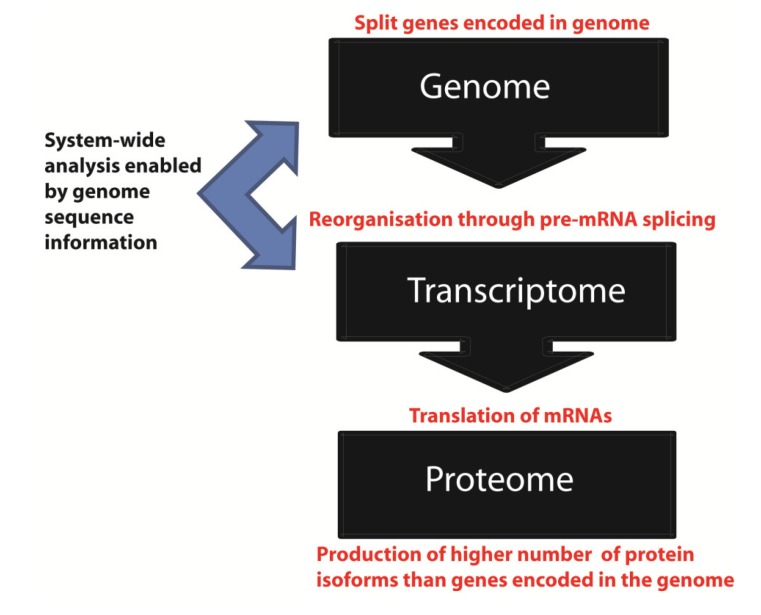
Information flow from the genome to the proteome. The genome represents an archive of information embedded in DNA. This information is transcribed as RNA to give the transcriptome, and then translated into protein to give the proteome.

The completion of the human genome sequence [1,2] brought together key scientific and philosophical questions, including exactly what we are as a species and individuals. However, in order to function, the genome has to be expressed. The primary expression product of the genome is RNA, and the complete set of all RNA molecules made through copying the genome into RNA is called the transcriptome (Figure 1). After transcription in the nucleus, mRNAs are translated into protein in the cytoplasm to yield the proteome while other RNAs have noncoding functions [3].

**Figure 2:**
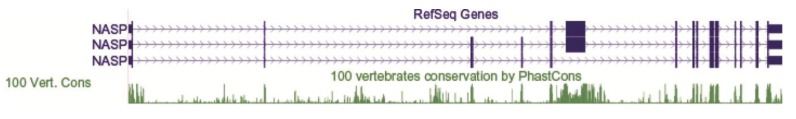
The intron-exon structure of a typical human gene displayed on the UCSC genome browser [6]. Introns are shown as lines (the “arrowheads” in the lines indicate the direction of transcription). Exons are shown as vertical bars. Coding exons are shown as thicker vertical bars than non-coding exon sequence. This example shows the *NASP* gene. The gene structures shown are “Refseq genes” that represent known human protein-coding and non-protein-coding genes taken from the NCBI RNA reference sequences collection. Notice that this single gene locus contains three distinct Refseq annotations containing different exon structures. Conserved sequences detected by comparative genomic information from 100 vertebrate genome sequences are shown at the bottom as a Phastcons plot—the higher values are most conserved, and often correspond to exons.

A key feature of human (and other eukaryotic) genes is their split exon-intron structure [4,5]. Figure 2 shows the exon-intron structure of a typical human gene displayed on a genome browser [6]. The exons include the protein coding information of the gene while introns are the intervening sequences between them. The term exon refers to the fact that exon sequences are expressed in the mRNA made from the gene, as opposed to introns which are removed (intron refers to intragenic regions) [7]. The presence of introns within genes and the long intergenic sequences between genes mean that only a small fraction of the human genome is truly protein-encoding. To put some figures on this, human protein-encoding genes contribute ~33.5% of the human genome sequence [1,2] but exons alone comprise 2.94% of the genome [8]. Protein coding exons make up a smaller proportion still (1.2%) of the genome. This is because there are at least partially untranslated exons in every mRNA (some of which can have important regulatory roles), and some exons remain entirely untranslated (see below).

Genes need to be transcribed over their full length (including both introns and exons) to generate precursor mRNAs (pre-mRNAs). Transcription of long genes represents a considerable energy investment by the cell. One of the longest genes in the human genome, *DYSTROPHIN* takes in the order of 16 hours to transcribe, yet produces a final mRNA of just ~14 kb that would have just taken ~7 minutes to transcribe by itself, assuming an elongation rate of 2 kb/minute [9]. It is calculated that ~95% of RNA does not leave the nucleus [10]. Nuclear-retained RNA includes intron sequences and some long ncRNAs that are also spliced but retained in the nucleus.

The splicing reaction is catalysed by the spliceosome. While introns are generally discarded after splicing, some introns can yield functional RNAs after splicing (e.g., miRNAs) [11,12]. Spliceosomes themselves are multi-component machines containing five snRNAs and at least 200 proteins, making them one of the most complicated assemblies in the cell [13,14]. Spliceosomes assemble *de novo* around each intron to be removed. A typical gene containing eight exons would require the assembly of eight spliceosomes to create a functional mRNA. Input of energy is required for spliceosomes to properly assemble through multiple ATP-dependent RNA helicases and other energy consuming proteins (including GTPases) [15].

Prior to completion of the human genome sequence, research into splicing typically looked at single genes and exons as models. However, while these detailed studies continue to be very important, the advances in genomics catalysed by genome sequencing projects have spawned parallel advances in transcriptomics, enabling a much broader system-wide dissection of RNA processing pathways. Here I review some of these new insights. While the focus here is on pre-mRNA splicing, transcriptome-wide analyses have also been directed at other aspects of genome expression, including RNA editing, RNA stability, expression of ncRNAs, polyadenylation and translation.

## 2. The Human Genome Sequence Has Led to New Global Insights into the Control of Splicing

In the 1980s examination of a limited number of genes led to the identification of short conserved sequences called 5' and 3' splice sites at exon-intron junctions [16]. The availability of the human and other genome sequences have enabled these studies to be extended genome-wide [17]. Most human exons are spliced together by a single kind of major spliceosome that recognises most 5' and 3' splice sites. In addition a second minor spliceosome exists in parallel that decodes a smaller subset of intron–exon junctions [18]. This minor spliceosome has a different but overlapping complement of snRNAs to the major spliceosome. Recent transcriptome-wide data show a key snRNA component in the minor spliceosome (called U6_ATAC_) is an important gene expression switch controlling patterns of splicing [19]. In the rest of this review the activities of the major and minor spliceosomes are not separately distinguished.

The conserved 5' and 3' splice site sequences encoded in the genome at exon-intron junctions are quite short. Furthermore, scattered within introns are short sequence elements called pseudoexons. Pseudoexons “look like” exons in that they have 5' and 3' splice sites, but are not selected as exons by the spliceosome. Estimates from model human genes suggest pre-mRNA splicing is remarkably accurate [20]. However, transcriptome-wide analyses of splicing patterns using RNAseq do detect some errors in splicing (at a rate of around 0.7% errors/intron)—these errors have been termed “noisy splicing”, and might contribute to gene and protein evolution by enabling new mRNA isoforms to be made even at low frequencies [21].

The spliceosome uses several mechanisms to accurately decode exon/intron structure using information embedded in the transcriptome. Firstly, in humans and most vertebrates, spliceosomes recognise exons from introns through a process called exon definition [22,23,24]. The advantage of exon definition is that since exons are quite small they should be easier to identify as discrete units compared most (considerably longer) vertebrate introns. In exon definition, early spliceosome components bind to the pre-mRNA and “flag” exons to be spliced together. Following exon definition, pairs of exons are then joined together by splicing which removes the intervening intron sequences. Amongst the early binding components of the spliceosome involved in exon definition are the U1 snRNP RNA-protein complex that recognises the 5' splice site, and a protein dimer which recognises 3' splice sites called U2AF (U2AF65 and U2AF35 are the two proteins in the dimer).

A second mechanism that facilitates accurate decoding of the genome is the presence of a splicing code that helps to differentiate between exons and introns in pre-mRNA. Before the sequencing of the human and other eukaryotic genomes, the important sequences controlling splicing of an exon were usually worked out on a gene by gene basis, using a finite number of model exons. The importance of exon sequences outside the splice junctions for splicing were first identified in pioneering experiments using model exons in the *FIBRONECTIN* and β globin genes [25,26]. It is now known that pre-mRNAs each contain multiple short nucleotide sequences that can enhance or silence splicing of their associated exons [27,28,29]. Exon sequences can function as exonic splicing enhancers (abbreviated ESEs) that help the spliceosome to recognise exons, or exonic splicing silencers (abbreviated ESSs) that inhibit spliceosome recognition by the spliceosome. Similarly, intron associated sequences can function as intronic splicing enhancers (abbreviated ISE) or Intronic Splicing Silencers (abbreviated ISSs). Splicing enhancers are bound by proteins or complexes of proteins, including the SR proteins that contain domains enriched in serine and arginine residues, and splicing silencers are frequently bound by heterogeneous ribonucleoproteins (abbreviated hnRNPs).

**Figure 3:**
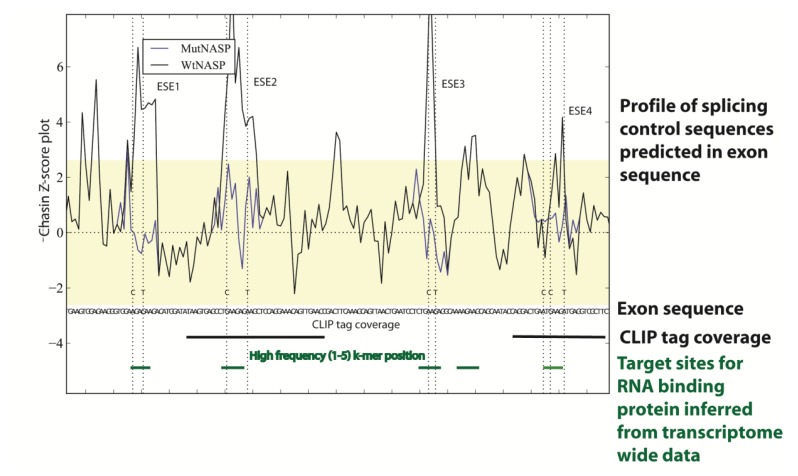
Transcriptome-wide data can be used to predict the splicing code in specific genes. In this example sequences within a cassette exon in the mouse NASP gene have been analysed using genome and transcriptome wide datasets to pinpoint splicing control sequences. Firstly a Chasin Z-score plot was used that can identify sequences predictive of exonic splicing enhancers and silencers [32,34]: the four exonic splicing enhancer (ESE) sequences identified are shown as peaks in the plot above the sequence. In this example, these ESE sequences were individually mutated to test function in minigenes (the Chasin profiles of the mutants M1-M4 are shown compared to the wild type sequence: notice the change in the Z-score plot removes predicted ESE activity for each mutant). The positions of these ESEs mapped to binding sites for the splicing factor Tra2β, both individually identified using cross linking immunoprecipitation (CLIP) in the mouse testis and predicted from the *in vivo* binding site generated from transcriptome-wide Tra2β binding data from the mouse testis. This figure is adapted from [32].

The availability of genome sequences have allowed system-wide approaches to identify splicing enhancers and silencers that control splicing and led to significant insights into the “splicing code” [29]. These approaches have included machine learning approaches to utilize hundreds of features in pre-mRNAs including motifs bound by RNA binding proteins and RNA secondary structure predictions to predict *in vivo* splicing decisions [30]. Computer programmes have also been devised that can computationally predict positions of predicted splicing enhancers and silencers and the target sequences of RNA binding proteins in an input genomic sequence (e.g., Figure 3) [30,31,32,33]. The combination of these system-wide experimental and bioinformatic analyses show the splicing code is maintained as nucleotide information in the genome. The splicing code has similar importance to the genetic code that is deciphered to read amino acid sequences from mRNAs. However, the splicing code is much more complex than the genetic code. While the genetic code uses triplet codons to specify amino acids, in the splicing code multiple sequence elements act in combination to decipher the exon/intron structure of pre-mRNAs [30].

**Figure 4:**
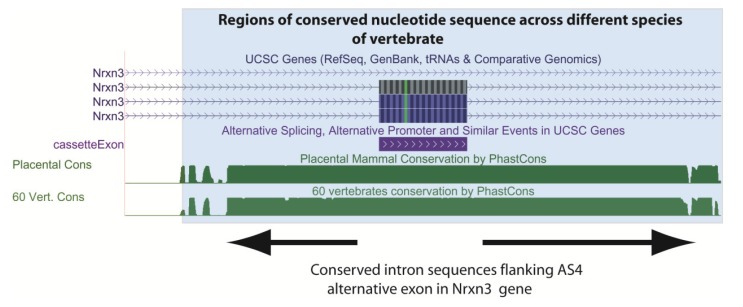
A functional requirement to maintain splicing control sequences constrains evolution of the genome. This screenshot is downloaded from the UCSC genome browser [6] and shows conserved intron sequences flanking the alternatively spliced AS4 exon from the mouse *Nrxn3* gene. The conserved sequences are shaded. At the top, the UCSC gene annotations show that this cassette exon is included in ¾ mRNA isoforms made from this gene. At the bottom the Phastcons plot shows that the flanking intron sequences are also highly conserved as well as the exon sequence (exons might be conserved because of their protein-coding content). Conservation of these intron sequences are likely important to control tissue-specific splicing of this exon by the spliceosome. Known alternative events are annotated on the UCSC track “Alt events”, and can be shown alongside the gene structure (here the cassette exon is annotated in the alt events track, and is in purple).

**Figure 5:**
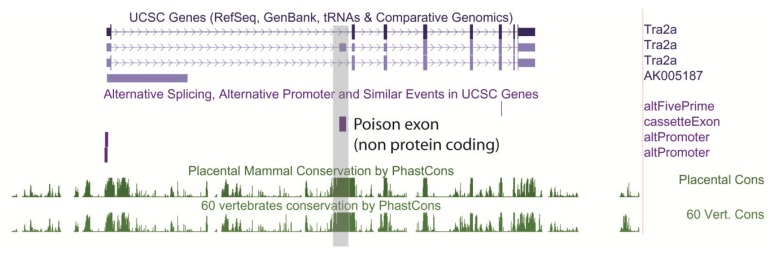
Non protein coding exons are conserved in the genome. Some non-coding exons are highly conserved in the human genome and play important roles in controlling the expression levels of splicing regulator proteins. The *TRA2A* gene encodes the splicing regulator protein Tra2α and contains a poison exon that contains multiple stop codons and is only inserted into some mRNAs. Despite not containing coding information, the *TRA2A* poison exon is highly conserved across species (indicated by the Phastcons score). Notice that the TRA2A gene encodes also alternative 5' splice sites and uses alternative promoters. This screenshot was downloaded from the UCSC genome browser [6].

Because they are needed for exons to be spliced into mRNAs, ESE sequences have been maintained in exons as well as the codons that specify amino acid sequences [35]. The intronic sequences that flank exons are often also strongly conserved between species (Figure 4 shows as an example conserved nucleotide sequences flanking an exon in the mouse *Neurexin3* gene downloaded from the UCSC genome browser). Comparison of the human and mouse genomes [1,36] which diverged 75 million years ago show that alternative exons are usually flanked by much longer stretches of conserved intron sequences than constitutive exons, consistent with more elaborate control mechanisms [37,38]. Conservation in these exon flanking intron sequences in some cases is much higher than in promoters, suggesting that one of the main functions of conserved noncoding sequences between mouse and human is the regulation of alternative splicing [37]. Even non-protein coding exons can be highly conserved in the genome. Noncoding exons include highly conserved “poison exons” (for example see Figure 5), that when included insert premature translational termination codons and lead to mRNA decay [32,39]. “Poison exons” are very important for auto-regulation of RNA binding proteins that control splicing [39,40]. Together these studies show the maintenance of splicing control sequences has had a significant impact in constraining genome evolution.

Genome sequences have been used to help develop technologies aimed to globally dissect RNA processing pathways [41]. These technologies can identify the target sites of RNA binding proteins transcriptome-wide. In cross linking immunoprecipitation (abbreviated CLIP) experiments RNA binding proteins are cross-linked *in situ* to their target RNAs within intact cells using ultra violet irradiation, followed by immunoprecipitation of the RNA protein complexes and amplification by PCR [42,43,44]. Once unique target sites are identified by next generation sequencing (these are called CLIP tags), these can be mapped onto genome sequences to reveal the initial binding sites in the transcriptome. Transcriptome-wide CLIP analyses have enabled maps to be drawn of the target sites of RNA binding proteins relative to regulated exons, and these maps can be used to predict mechanisms of splicing control [45]. For example, CLIP tags of the RNA binding protein Tra2β that is needed for splicing inclusion for a regulated cassette exon in the NASP gene are shown in Figure 3 [32].

The completion of the human genome sequence enabled the development of comprehensive microarrays to interrogate gene expression. These global techniques include the development of splice-sensitive microarrays. These microarrays either detect specific exons in the transcriptome or the use of specific splice junctions and report splicing patterns in mRNA [46,47]. Transcriptome-wide patterns of alternative splicing can also be detected by RNAseq [48,49]. These technologies have been used to search for exons mis-spliced after depletion of particular RNA binding proteins from cells. By analysing thousands of exons in parallel the splicing events that are regulated by specific RNA binding proteins can be comprehensively identified. These experiments have shown some individual RNA binding proteins bind to and regulate similar mRNAs. For example, the NOVA protein regulates splicing of a functionally coherent set of genes involved in synapse function [50]. Other splicing regulators might similarly have coherent RNA functional targets, including T-STAR and SAM68 which regulate regional alternative splicing of the synaptic neurexin genes in the brain [51,52].

Knowing which splicing events are regulated by what proteins at a global level has been used to derive general rules. For example, binding of the NOVA proteins upstream of exons tends to block splicing, while binding of the same proteins downstream of exons enhance splicing [44,45]. These rules governing the RNA motifs bound by NOVA and their role in activating or repressing associated exon inclusion are conserved between flies and mammals, although the actual target mRNAs are different [53]. Similar rules have also been uncovered for PTB and some other splicing regulator proteins [45,54,55]. Recent developments to understand the splicing code have compared binding maps for different RNA binding proteins, and show that some functionally collaborate with each other to generate tissue specific splicing patterns [56].

Transcriptome-wide insights have have also revealed the involvement of RNA binding proteins in other aspects of genome biology. Alu sequences are retro-transposable elements that frequently insert into introns, and have sequence similarities to exons [57]. There are over 15,000 Alu sequences in the human genome, many of which are inserted into introns. The RNA binding protein hnRNP C has an important role for in protecting the transcriptome from potential mis-splicing caused by the insertion of Alu retrotransposable elements into genomic introns [58]. Depletion of hnRNPC leads to aberrant inclusion of around 1000 Alu-derived exons into the transcriptome. Alu sequences contain polypyrimidine tracts that potentially bind U2AF65, leading to them being aberrantly included into mRNAs, but this splicing is blocked by hnRNPC.

## 3. Analysis of the Human Transcriptome Led to the Realisation That Most Human Genes Encode Alternatively Spliced mRNAs

**Figure 6:**
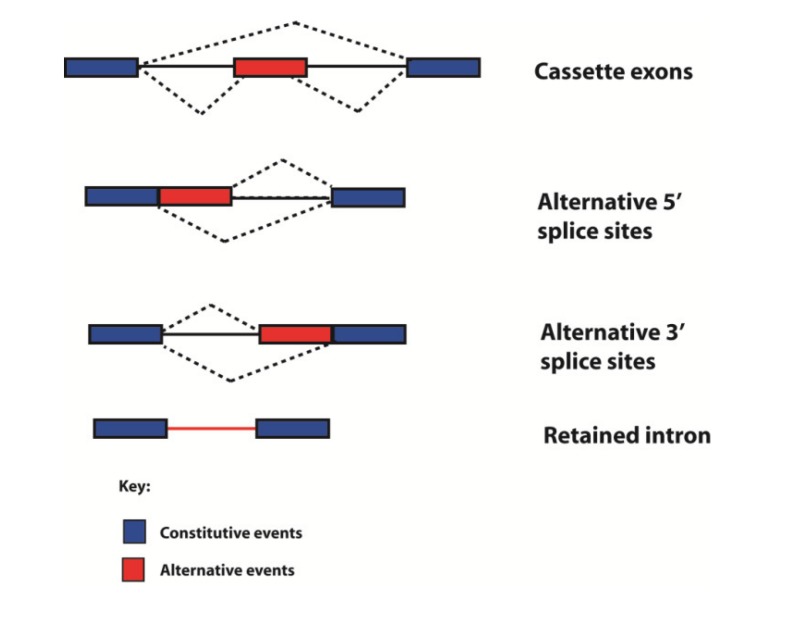
Types of alternative splicing events detected in the human transcriptome. Exons are shown as boxes, introns as lines, and splicing patterns as broken lines. mRNAs can be made from individual genes can using multiple alternative events, including different types of splicing, to build up complex patterns of alternative spliced mRNAs.

Historically genes have been defined in different ways by different people at different times. Following the human genome sequencing project, the fundamental definition of what a gene actually is has been enriched, and to some extent clouded, by comparison of genome and transcriptome sequences [59,60]. Alternative mRNA isoforms can originate from the same genetic loci through use of alternative promoters and polyadenylation sites, and by the process of alternative splicing through which exons can be spliced into different combinations to give multiple mRNAs. Hence a “single gene” can encode multiple products. Alternative splicing fits into four different categories, depending on how variable splice junctions are utilised (Figure 6). In the simplest form of alternative splicing, called exon skipping, whole exons are either spliced into the mRNA or skipped (ignored by the spliceosome). Exon sequences can also be spliced into mRNA using different splice sites (alternative 5' splice site or 3' splice sites can be selected). Whole introns can also be left in the mRNA (this is called intron retention). Transcriptome-wide analyses show that in humans exon skipping is the most frequent form of alternative splicing, and intron retention the least frequent [61].

Evidence provided by comparative genome and transcriptome sequences has shown alternative splicing to be extremely frequent. Before the human genome was published, random sequencing of human mRNAs from their 3' ends using oligo dT priming suggested ~40% of human genes encoded alternatively spliced mRNAs [62,63]. During the human genome sequencing project, reconstruction of mRNAs from the gene rich chromosomes 19 and 22 upped this estimate to 59% of genes encode alternative mRNAs, with 2–3 mRNA isoforms made/gene [1]. The use of microarrays to detect global patterns of alternative splicing indicated 73%–74% of human genes express alternatively spliced mRNA isoforms [46,64]. The most recently reported RNAseq analyses of the human transcriptome is consistent with ~95% of multi-exonic genes expressing variant mRNA isoforms, with a plateaux of 10–11 isoforms/gene/cell line [65]. Usually one or two major mRNA isoforms predominate in a given cell line so many cell types will just express one major mRNA isoform [60,65,66]. 86% of genes have a minor isoform frequency of 15% or more, and more than 50% of alternative exons are tissue specific in expression [48,67]. Alternative events are now annotated on genome browsers like the UCSC genome browser (e.g., Figure 4) [6].

## 4. To What Extent Can Human Complexity Be Ascribed to Alternative Splicing?

Proteins make up large components of human bodies. Prior to the genome sequence estimates of human gene numbers went as high as 100,000. An initial surprise from the human genome sequence was a much lower protein coding gene number, initially counted at around 23,000 [1,2]. The most recent gene counts from the ENCODE consortia suggest only 20,687 human protein coding genes [8]. The number of proteins expressed in a human cell is in contrast estimated to be ~100,000 [68,69]. This protein number represents an amplification factor of 5-fold compared to the counted number of genes. This total gene number in humans does not seem to be exceptionally higher than seemingly less sophisticated organisms. The genome of *Haemophilus* *influenza* contains 1743 predicted genes, the yeast *Saccharomyces cerevisiae* ~6000 genes, *Drosophila melanogaster* ~13,600 genes, and nematode worms 18,425 genes [70,71,72]. 

**Figure 7:**
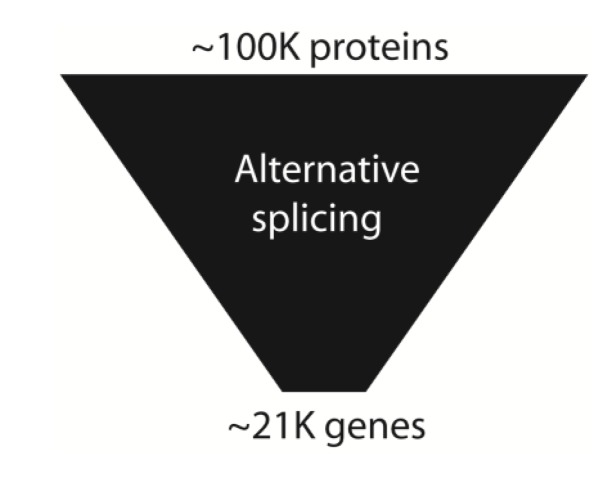
Alternative splicing amplifies genome information. Alternative splicing amplifies information in the human transcriptome relative to the genome.

This apparent failure of gene numbers to correlate with complexity has been called the gene number paradox, and counted as one of the major surprises from the human genome sequence. To what extent might alternative splicing and the resulting expansion in protein coding information help explain developmental and physiological complexity in humans (Figure 7)? Until recently this question has been difficult to address, since the higher number of mRNA and EST sequences available from humans made comparisons of alternative splicing frequency with other species biased. However, a recent modencode project based on RNAseq analysis to look at alternative splicing in *C. elegans* found ~25% alternative splicing in 5000 genes, with around 30% of these being alternatively spliced between different developmental stages [73]. In depth experiments using RNAseq and tiling arrays do show a lower frequency (60.7%) of genes in the fruitfly are alternatively spliced than in humans, often in a developmental or sex-specific fashion [74].

Hence amongst multicellular animals investigated in detail, humans do seem to exhibit higher levels of alternative splicing, which might lend credence to the idea that alternative splicing may be a factor contributing to human sophistication. If phylogenetic differences in alternative splicing frequency correlate with complexity, one might expect a decreased level in single celled organisms compared with multicellular organisms. On the one level less introns are found in the single celled baker’s yeast *Saccharomyces cerevisiae*: only 5% of genes contain introns in this yeast (290/6000 genes). However, these lower intron numbers are a bit misleading. The reason for a low overall intron number in this yeast is that many introns have been lost because of reverse transcriptase activity converting mRNAs into cDNAs, which then re-integrate into the genome through high levels of homologous recombination replacing originally intron containing genes [75]. While intron-containing genes generally are rare in yeast, alternative splicing of some of these introns are specifically utilised to control developmental timing of during meiosis that takes place under conditions of limiting nutrients [76,77] so alternative splicing is used to control a complex stage in the lifecycle of this single celled organism. Taken as a whole, it is difficult to draw general correlations between overall frequencies of alternative splicing and organism sophistication.

Alternative splicing patterns can also evolve rapidly and sometimes differ between closely related species. For example, despite almost 99% identity in protein coding information, comparative transcriptome analyses have shown that 6%–8% of alternative spliced exons have different inclusion patterns between humans and chimps. These observations are consistent with alternative splicing contributing to species-species differences, and transcriptomes being more distinct between species than protein coding information [78]. Although the major conclusion from evolutionary comparisons is that much alternative splicing is not conserved between species, comparative genomics show some alternative exons have been highly conserved during evolution [79,80,81]. These include the highly conserved “AS4” alternative exon in the *Neurexin3* gene (abbreviated *Nrxn3*, Figure 4) that is conserved across the vertebrate lineage [51]. The *Neurexin* genes have been linked with autism and schizophrenia, and mice genetically engineered to be unable to regulate this AS4 alternative exon in the *Nrxn3* gene have different synaptic activity in the brain [82].

Post-genome analyses have also addressed the question what alternative splicing does. Protein sequences encoded by alternatively spliced exons are frequently involved in protein-protein interaction networks and contain signalling domains [83,84]. Some alternatively spliced mRNAs encode proteins with clearly different functional activity. For example, different mRNA splice isoforms encoding the FOXP1 transcription factor are made between neural stem cells and differentiated cells, and these encode proteins that activate different promoters [85]. Alternative splicing regulators have been implicated in human cognitive diseases like autism [86]. Different groups of genes are regulated by alternative splicing from those regulated by transcription [87].

Alternative splicing pathways have been shown to have important roles in controlling development, and some human diseases are caused by defects in alternative splicing including the multi-system disorder myotonic dystrophy [87]. However, individual RNA binding proteins likely regulate coordinated splicing programmes of many target exons, and each of these individual exons might only have somewhat subtle biological contributions. Furthermore, post-genome technologies are also starting to introduce a note of caution in the interpretation of high levels of alternative splicing. Some lower abundance splice variants might be non-functional isoforms that occur as a result of low frequency events mistakes in the splicing process if they are neither evolutionarily conserved nor protein-coding [21,88]. Hence the frequency of functional alternative splicing is likely to be lower than the total frequency of all alternative splicing events.

## 5. Human Genome Packaging into Chromatin Correlates with Its Intron/Exon Structure

Post genome analyses have shown that the genome and transcriptome are intimately linked. In particular the exon/intron structure of genes correlates with how the genome is packaged. Within the nucleus the genome is wrapped around protein complexes called nucleosomes to form chromatin. Each nucleosome is itself made up of eight positively charged histone molecules that can be modified by the addition of small chemical groups (typically methyl and acetyl groups) [89]. Packaging of the genome within chromatin is important to enable storage of chromosomes within the comparatively small space afforded by the nuclear volume. 

After experimental treatment of chromatin with the enzyme DNAse I, the DNA sequences wrapped around nucleosomes remain protected, while the DNA linkages joining nucleosomes together become degraded. Genome-wide analysis of sequences protected from DNAse I digestion in humans and other species indicate a 1.5 fold enrichment of exon sequences over nucleosomes compared to intron sequences [90,91]. This means that in chromatin exons are preferentially (but not exclusively) associated with nucleosomes. This is likely to have a biochemical explanation: exon sequences are GC-enriched, while their flanking intron sequences are AT-rich. Nucleosomes interact more strongly with GC-rich sequences, which likely help anchor exons to nucleosomes. The association of exons with nucleosomes has in turn had important implications for genome evolution. A length of ~150 nucleotides of DNA is needed to wrap around a nucleosome, and this is also the average size of an exon. Hence nucleosome wrapping has placed an evolutionary constraint on exon size, while in contrast introns have been able to expand in size to thousands of nucleotides.

## 6. Most Splicing Occurs Co-Transcriptionally

Another important connection between the genome and the transcriptome is their physical proximity during important RNA processing steps. In several species including humans much pre-mRNA splicing has been shown to take place co-transcriptionally [92]. This means that “full length” pre-mRNA copies of genes are not made. Instead processing takes place as pre-mRNAs are produced on nascent pre-mRNAs still attached to RNA polymerases engaged in transcription. Deep sequencing of fractionated nascent RNA in human cell lines and total RNA in the brain show that exons located more upstream in genes are most likely to be spliced on nascent RNA [65,93,94]. Transcription of the genome also functionally depends on components more “traditionally” thought to be involved in splicing. Transcriptome-wide analysis of gene expression following depletion of U1 snRNP (a component of the spliceosome that recognises 5' splice sites) has shown that high nuclear concentrations of U1 snRNP are needed to prevent premature intragenic polyadenylation at sites upstream of the proper 3' boundary of genes [95].

The fact that splicing takes place on chromatin during ongoing transcription has important implications for alternative splicing patterns. Single molecule experiments have shown that nucleosomes slow down the progress of transcription [96,97]. This means that the time taken to traverse nucleosomes can provide pauses in RNA polymerase II elongation, allowing the spliceosome a window to assemble on nascent pre-mRNA. Nucleosomes have thus been described as “speed bumps” [98,99,100,101]. Interestingly only true exons, and not pseudoexons, are associated with nucleosomes [90,91]. Exon association with nucleosomes is likely to help in their recognition by the spliceosome. Pauses in transcriptional elongation on bona fide exons might facilitate spliceosome assembly before potentially competing splice sites in downstream pseudoexons can be transcribed. Changes in chromatin structure can locally speed up or slow down transcription within genes and be important for controlling alternative splicing [99]. Local transcriptional pauses would provide spliceosomes a longer “window” of time in which to assemble and sometimes even carry out splicing of an exon before a competing downstream splice site appears [102,103]. Faster elongating RNA polymerase II enzymes would give spliceosomes less time to assemble on nascent pre-mRNA before competing exons were transcribed, and so would favour exon skipping. In some cases changes in histone modification can recruit or stabilise RNA binding proteins which regulate splicing of the pre-mRNAs made from the gene [104]. The interactions between the different processes in gene expression have been recently reviewed in [105].

## 7. Conclusions

The human genome sequence has provided a catalyst for understanding the transcriptome. System-wide approaches of the transcriptome have led to a fuller appreciation of how genomes work including how the human genome operates with a finite gene number and providing a system wide view of RNA processing.
